# Increased Expression of Orexin-A in Patients Affected by Polycystic Kidney Disease

**DOI:** 10.3390/ijms25116243

**Published:** 2024-06-05

**Authors:** Ersilia Nigro, Daniela D’Arco, Fiorenzo Moscatelli, Antonio Pisani, Maria Amicone, Eleonora Riccio, Ivana Capuano, Francesca Argentino, Marcellino Monda, Giovanni Messina, Aurora Daniele, Rita Polito

**Affiliations:** 1 Dipartimento di Scienze e Tecnologie Ambientali, Biologiche, Farmaceutiche, Università della Campania “Luigi Vanvitelli”, Via Vivaldi 43, 81100 Caserta, Italy; ersilia.nigro@unicampania.it; 2 CEINGE-Biotecnologie Avanzate Scarl “Franco Salvatore”, Via G. Salvatore 486, 80145 Napoli, Italy; darco@ceinge.unina.it (D.D.); francesca.argentino@ceinge.unina.it (F.A.); 3 Department of Human Sciences, Telematic University Pegaso, 80100 Naples, Italy; fiorenzo400@gmail.com; 4 Unità di Nefrologia, Dipartimento di Sanità Pubblica, Università di Napoli “Federico II”, Via Pansini 5, 80131 Napoli, Italy; antonio.pisani13@gmail.com (A.P.); ma.amicone.90@gmail.com (M.A.); eleonora.riccio@unina.it (E.R.); ivana.capuano@unina.it (I.C.); 5 Sezione di Fisiologia Umana e Unità di Dietetica e Medicina dello Sport, Dipartimento di Medicina Sperimentale, Università degli Studi della Campania “Luigi Vanvitelli”, 80138 Naples, Italy; marcellino.monda@unicampania.it (M.M.); giovanni.messina@unicampania.it (G.M.); 6 Dipartimento di Medicina Molecolare e Biotecnologie Mediche, Università degli Studi “Federico II”, Via Pansini 5, 80131 Napoli, Italy; 7 Department of Clinical and Experimental Medicine, University of Foggia, 71122 Foggia, Italy; rita.polito@unifg.it

**Keywords:** orexin-A, polycystic kidney disease, blood pressure, renal function, marker

## Abstract

Orexin-A is a neuropeptide product of the lateral hypothalamus that acts on two receptors, OX1R and OX2R. The orexinergic system is involved in feeding, sleep, and pressure regulation. Recently, orexin-A levels have been found to be negatively correlated with renal function. Here, we analyzed orexin-A levels as well as the incidence of SNPs in the hypocretin neuropeptide precursor (HCRT) and its receptors, HCRTR1 and HCRTR2, in 64 patients affected by autosomal dominant polycystic kidney disease (ADPKD) bearing truncating mutations in the *PKD1* or *PKD2* genes. Twenty-four healthy volunteers constituted the control group. Serum orexin-A was assessed by ELISA, while the SNPs were investigated through Sanger sequencing. Correlations with the main clinical features of PKD patients were assessed. PKD patients showed impaired renal function (mean eGFR 67.8 ± 34.53) and a statistically higher systolic blood pressure compared with the control group (*p* < 0.001). Additionally, orexin-A levels in PKD patients were statistically higher than those in healthy controls (477.07 ± 69.42 pg/mL vs. 321.49 ± 78.01 pg/mL; *p* < 0.001). Furthermore, orexin-A inversely correlated with blood pressure (*p* = 0.0085), while a direct correlation with eGFR in PKD patients was found. None of the analyzed SNPs showed any association with orexin-A levels in PKD. In conclusion, our data highlights the emerging role of orexin-A in renal physiology and its potential relevance to PKD. Further research is essential to elucidate the intricate mechanisms underlying orexin-A signaling in renal function and its therapeutic implications for PKD and associated cardiovascular complications.

## 1. Introduction

Polycystic kidney disease (PKD), one of the most prevalent genetic kidney diseases, is typified by the bilateral development of cysts, fluid-filled sacs, which have the potential to progressively replace a large portion of healthy kidney tissue, decreasing kidney function [1]. In addition, the presence of these cysts can impede urine flow, elevate blood pressure, and increase the risk of urinary tract infections [2]. PKD, encompassing autosomal dominant (ADPKD) and recessive (ARPKD) forms, along with atypical variations, not only leads to renal complications but also induces extrarenal issues such as hypertension and cardiovascular ailments [3,4]. Data from the literature shows that approximately 50% of patients with ADPKD progress to end-stage renal disease by age 60. ADPKD is essentially due to mutations in the PKD1 and PKD2 genes. PKD1 encodes polycystin 1 (PC1) and PKD2 encodes polycystin 2 (PC2). PC1 and PC2 proteins interact to regulate cell proliferation, cell migration, and cell–cell interactions, playing a key role in the pathogenesis of ADPKD disease [1,3,4]. In particular, approximately 80% of patients with ADPKD have mutations in PKD1, while 15% have mutations in the PKD2 gene; the remaining 5% of patients carry rare mutations in other genes [2,5,6,7,8]. Furthermore, PKD1 truncating mutations tend to hasten the progression to ESRD [8]. PKD also affects extrarenal organs, contributing to various complications such as liver cysts, brain aneurysms (in ADPKD), and heart valve abnormalities [9,10]. Different severity grades of the disease, at both the intra- and inter-familial levels, characterizes PKD patients, indicating a role of environmental factors and genetic background in determining primary symptoms and comorbidities [9,10]. Among these, hypertension is an early symptom of PKD progression that affects over 60% of patients and is influenced by numerous pathogenetic mechanisms that contribute to increasing morbidity and mortality from cardiovascular causes [10,11]. The variability in PKD severity underscores the importance of understanding its complex interplay between genetic background and environmental factors [9,10,11].

Orexin-A, also known as hypocretin-1, is a 33 amino acid neuropeptide, produced primarily in the lateral hypothalamus. It is one of two peptides derived from the same precursor protein (the other is orexin-B, or hypocretin-2). Orexin-A is primarily known for its role in promoting wakefulness and regulating sleep-wake cycles. It helps maintain arousal during wakefulness and is involved in stabilizing sleep-wake transitions. It also plays a role in the regulation of eating behavior and energy homeostasis. It stimulates food intake and is involved in reward-based eating behavior, and influences various autonomic functions, including the regulation of cardiovascular activity, thermoregulation, and gastrointestinal motility. Orexin-A exerts its effects by binding to and activating two G protein-coupled receptors: orexin receptor type 1 (OX1R) and orexin receptor type 2 (OX2R). These receptors are widely distributed throughout the brain and periphery. Orexin-A activation of orexin receptors leads to increased activity of the sympathetic nervous system, resulting in effects such as increased heart rate, blood pressure, and vasoconstriction. It has also been implicated in the regulation of stress responses, including the hypothalamic–pituitary–adrenal (HPA) axis, and in the modulation of stress-related behaviors. Furthermore, dysfunction of the orexinergic system is involved in various central nervous system disorders, including narcolepsy (characterized by excessive daytime sleepiness and cataplexy), insomnia, and mood disorders. In addition to its classical functions, orexin-A has been implicated in other physiological processes, including pain modulation, addiction, and cognitive function. Overall, this neuropeptide is multifunctional; it has various roles in regulating sleep-wake cycles, appetite, autonomic functions, stress responses, and other physiological processes. Its dysregulation or dysfunction may have implications for various neurological and metabolic disorders [12]. The orexinergic system contributes to the regulation of several physiological processes, including the central control of sympathetic nerve activity and blood pressure [13]. Accumulating evidence indicates that orexin-A is involved in sympathetic activation, thus increasing blood pressure levels [14,15,16]. Furthermore, some studies show that pharmacological blockage of orexin receptors is effective in reducing blood pressure [17]. The orexinergic system has been considered a link between obesity and hypertension since, in the Zucker rat model, obesity-related hypertension occurs through upregulation of the OX1R [18]. In addition, the orexinergic system has also been considered as a critical regulator of cardiovascular homeostasis [15].

Given the multifaceted roles of orexin-A, the orexinergic system has recently been found expressed in the kidney, and its levels have been found to be negatively correlated with renal function [19]. Furthermore, it has been demonstrated that orexin-A may regulate tubular function in the kidney through an autocrine, paracrine, or autocrine mechanism [19]. Genetic variants of orexin-A and, especially, of the OX1 and OX2 receptors have been associated with a predisposition for several metabolic and non-metabolic disorders [20]. Several single nucleotide polymorphisms (SNPs) in the *HCRT* gene have been studied [21], with some of them being associated with increased levels of orexin-A [22]. The *HCRTR1* and *HCRTR2* genes have been investigated for association with disorders like narcolepsy, Alzheimer’s disease, and headache [23]; in particular, a 408 isoleucine to valine mutation in the *HCRTR1* gene (rs2271933) is the most studied SNP [24].

Here, we analyzed orexin-A levels in 64 patients affected by PKD bearing truncating mutations in *PKD1* or *PKD2* genes and compared them to those in age-matched healthy subjects. Furthermore, we analyzed the incidence of SNPs in the gene encoding hypocretin neuropeptide precursor (HCRT) and its receptors *HCRTR1* and *HCRTR2* in PKDs. Correlation analysis was performed.

## 2. Results

### 2.1. Anthropometric, Biochemical and Clinical Features

The anthropometric, biochemical, and clinical features of the study participants are shown in Table 1. A total of 64 ADPKD patients and 24 healthy controls were recruited for this study. The chronic kidney disease (CKD) stages of the ADPKD patients were as follows: G1, 32.5%; G2, 21.6%; G3a, 17.6%; G3b, 17.6%; G4, 8.6%; and G5, 2.1%. The mean eGFR was 67.8 ± 34.53.

The study populations were comparable in terms of age, BMI, and sex distribution. Indeed, no statistical differences between controls and patients were found in relation to BMI and age. Lipid profiles (total cholesterol, triglycerides, LDL, and HDL) as well as glucose levels were comparable between patients and healthy subjects. Interestingly, systolic blood pressure was statistically higher in PKD patients (134.45 ± 15.14 vs. 122.76 ± 7.73, respectively; *p* < 0.009).

### 2.2. Orexin-A Levels Are Statistically Higher in PKD Patients Than Controls, Inversely Correlates with Blood Pressure and Directly with eGFR

**Figure 1:**
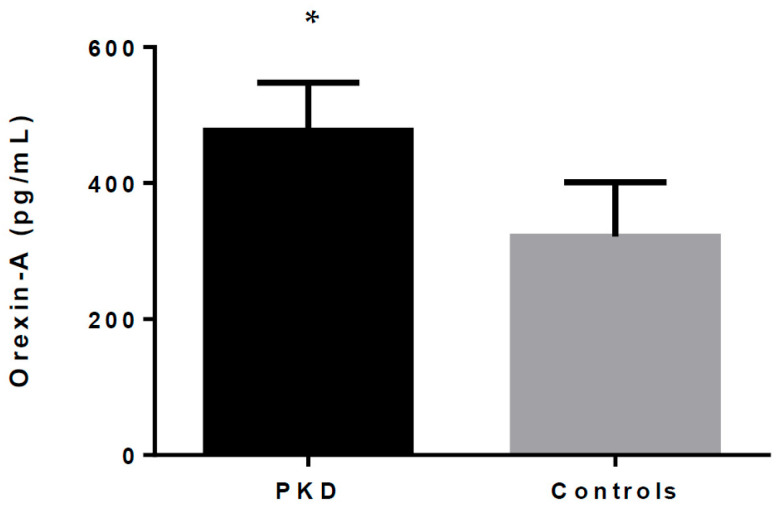
Orexin-A levels in PKD patients and healthy controls. * indicates *p* < 0.05.

As shown in Table 1 and Figure 1, orexin-A serum levels were statistically different between the patients and healthy controls. Our data show that total orexin-A levels in PKD patients were higher than in healthy controls (477.07 ± 69.42 pg/mL vs. 321.49 ± 78.01 pg/mL; *p* < 0.001).

**Figure 2:**
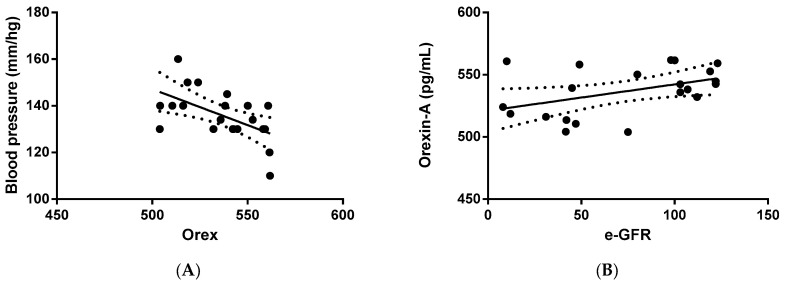
Correlation analysis between orexin-A levels and systolic blood pressure and eGFR in ADPKD patients. Orexin-A levels were found to be inversely correlated with blood pressure (mm/Hg) (**A**) and directly correlated with eGFR (mL/min/1.73 mq) (**B**). Solid lines represent the regression. The dotted line represents the average trend of the values (positive and negative).

Next, we verified whether orexin-A levels were correlated with clinical and/or biochemical parameters. The statistical analysis outlined that orexin-A was inversely correlated with blood pressure (*p* = 0.0085) in PKD patients (Figure 2A), suggesting an involvement of the protein in the regulation of arterial pressure. In addition, a direct correlation between orexin-A and eGFR was also found in PKD patients (*p* = 0.0462) (Figure 2B), implying the involvement of orexin-A in renal function.

### 2.3. HCRT SNPs

We analyzed the genotype of the *HCRT*, *HCTR1*, and *HCTR2* genes in people with ADPKD, investigating their distribution. Three ADPKD patients had one SNP (rs9902709) in the *HCRT* gene (see Table 2). No relevant values in orexin-A levels and clinical parameters were found in those patients, as reported in Table 2 (orexin-A mean value 458.11 ± 18.50 pg/mL). Notably, the rs9902709 SNP is located in intron 1 of the gene, a region which can regulate orexin-A serum levels; however, here, no difference was found, probably due to the small sample size.

The *HCTR1* and *HCTR2* SNPs were present in 51 and 59 PKD patients, respectively. The rs2271933 SNP in the *HCTR1* gene is located in exon 9, and is associated to the protein substitution p.(Ile408Val). The rs2653349 SNP in the *HCTR2* gene is located on exon 5, and implies the protein substitution p.(Ile308Leu).

## 3. Discussion

The lateral hypothalamus is the primary producer of the excitatory neuropeptide orexin-A [25]. Along with its well-established activities in the central nervous system related to controlling the circadian rhythm, there is mounting evidence that orexin-A is also involved in key processes in peripheral organs, including the kidney [26]. Among others, the function of orexin-A in the kidney is currently unclear, but the expression of OXR1 and OXR2 in the kidney [19,27], as well as orexin immunoreactivity in urine, strongly suggest a functional role for this peptide that results in involvement in a vast phenotypic spectrum of clinically and genetically heterogeneous disorders associated with a severe decline in kidney functionality [26]. The most frequent PKD forms are characterized by late onset and are typically due to mutations in both of the autosomal dominant genetically heterogeneous ADPKD causative genes, *PKD1* (approximately 78%) and *PKD2* (approximately 15%). Truncating mutations in both genes are associated with the most severe phenotype [5,6]. Cardiovascular abnormalities are common in patients with ADPKD, with hypertension being the most frequent finding, and are associated with a worse renal outcome [3]. Recently, we demonstrated that adiponectin could influence the clinical phenotype of ADPKD patients, and that to analyze the association between polymorphisms and ADPKD risk could potentially provide important insights into ameliorating kidney outcomes [28].

Here, we analyzed orexin-A serum levels in a cohort of patients with severe PKD caused by truncating mutations in *PKD1* and *2* genes, investigating biochemical and clinical parameters in comparison with healthy controls. The PKD patients were found to have significantly higher levels of orexin-A compared to healthy controls; to our knowledge, this is the first study considering PKD in particular in such a context. However, in accordance with our data, increased levels of orexin-A have also been found in different patients with renal function impairment [26].

In our sample of PKD patients, correlation analysis showed an inverse relationship between blood pressure and orexin-A levels. Orexin-A is involved in blood pressure regulation, as shown by functional in vitro and in vivo research. A number of studies using intracerebral injections of orexin-A have consistently concluded that orexin-A is a substance that raises blood pressure [29]. Additionally, the administration of agonists increases sympathetic activity and arterial blood pressure in normotensive animals, while transgenic orexin-A-deficient animals have lower resting blood pressure [30]. Nonetheless, an investigation carried out by Bastianini and associates discovered elevated blood pressure levels in mice without orexin [31]. There is a paucity of inconsistent clinical evidence about the connection between blood pressure or hypertension and the orexinergic system. Donadio et al. discovered that patients with orexin-A deficiency had lower blood pressure and a lowered heart rate when compared to healthy people, which is in contradiction to our data [32]. This is the first study to report a link between blood pressure and orexin-A in patients who do not experience changes in their sleep patterns; further research in a larger cohort is required to elucidate this relationship.

Furthermore, we discovered a direct correlation between orexin-A levels and eGFR. The link between plasma orexin-A levels and renal function (which exhibited a strong positive correlation with the serum albumin concentration and percent creatinine generation rate) was also observed by Sugimoto et al., which contradicts our findings. Nonetheless, they examined the plasma levels of orexin-A in hemodialysis patients and healthy individuals [33]. Following suit, Nakashima et al. examined older adults in general without hemodialysis and found the same outcomes, i.e., a negative connection between eGFR and plasma orexin-A levels [26]. Conversely, renal function was found to be inversely linked with the mRNA expression of orexin-A and its receptors (*HCRTR1* and *HCRTR2*) in peripheral blood cells by Kitajima et al. [34].

However, none of these published papers have analyzed PKD patients, and we cannot exclude that in such patients, additional mechanisms participate in the regulation of orexin levels that explain their direct relation with renal function.

Within Variation Viewer, SNPs are present in nearly half of the amino acids of orexin-A (15 out of 36 residues). Here we analyzed 19 variants in HCRT gene (Supplementary Table S1). Three PKD patients bore the rs9902709 SNP in the HCRT gene. This SNP has been previously found in obstructive sleep apnea syndrome, where it causes up to 1.5-fold increases in sera levels of orexin-A [22].

Another term for polymorphism-carrying SNPs is the genes encoding for the receptors HCRTR1 and 2. The most significant variant in HCRTR1, rs2271933, is a possibly functionally significant C/T mutation in exon 7 that results in an amino acid switch from isoleucine to valine (Ile408Val). It is suggested to augment OX1 receptor signaling (for example, through increased expression or decreased cycling/desensitization), consequently elevating the orexinergic tone. Regarding the HCRTR2 gene, the most studied variant is the rs2653349 SNP, which has been linked primarily to cluster headaches [35]. The more common wild type G-allele has been linked to increased risk, while the less common mutated A-allele reduces cluster headache risk. Indeed, studies analyzing Italian and German patients revealed that the G/G genotypes were at increased risk for the disorder, compared with A/G and A/A genotypes [36,37]. Here, we only found one ADPKD patient wt for the rs2653349 SNP, and therefore it is quite difficult to conclude whether an association might exist between this SNP and cardiovascular event predisposition in ADPKD.

Overall, several studies have suggested that orexin-A may influence kidney function through its effects on nervous system activity and blood pressure regulation. Activation of orexin-A receptors, particularly orexin receptor type 1 (OX1R), has been shown to increase sympathetic outflow, leading to vasoconstriction and alterations in renal blood flow and glomerular filtration rate (GFR). Furthermore, orexin-A may also exert direct effects on renal tubular function. Some studies have reported the presence of orexin-A receptors in renal tubular cells, suggesting a potential role in the regulation of electrolyte and water balance. However, the exact mechanisms underlying the effects of orexin-A on renal function are not yet fully understood, and further studies are needed to clarify its role in renal physiology and pathophysiology, particularly in ADPKD. Furthermore, it is important to consider that the effects of orexin-A on renal function may be influenced by various factors, including its interactions with other neurotransmitter systems and hormonal regulation. In summary, while orexin-A is primarily known for its role in regulating sleep-wake cycles and energy balance, emerging findings suggest that it may also play a role in modulating renal function through its effects on renal activity and the sympathetic nervous system, and potentially its direct actions on renal activity and renal tubular cells.

The field holds promise for the development of novel drugs targeting the cardiovascular system in ADPKD. Currently, there is a lack of data regarding the effects of pharmacological interventions aimed at modulating orexin-A’s influence on arterial pressure. However, molecules targeting orexin receptors, particularly antagonists, are already utilized for treating insomnia. These antagonists fall into two categories based on their mode of action on either one or both orexin receptors: single orexin receptor antagonists (SORAs) and dual orexin receptor antagonists (DORAs). While the potential of orexin-A as a target for pharmacological therapies in controlling blood pressure for ADPKD patients is intriguing, further translational studies are necessary to confirm its efficacy.

In conclusion, our analysis sheds light on orexin-A’s potential involvement in renal physiology, particularly in the context of severe PKD. Patients with PKD displayed significantly elevated levels of orexin-A compared to healthy controls, suggesting a potential link between this neuropeptide and renal dysfunction. Notably, inverse associations were observed between orexin-A levels and blood pressure, as well as positive correlations with eGFR. These findings align with previous research indicating orexin-A’s involvement in blood pressure regulation and renal function modulation. In summary, our study highlights the emerging role of orexin-A in renal physiology and its potential relevance to PKD. Further research is essential to elucidate the intricate mechanisms underlying orexin-A signaling in renal function and its therapeutic implications for PKD and associated cardiovascular complications.

## 4. Materials and Methods

### 4.1. Study Participants

Sixty-four patients affected by ADPKD were recruited from the Nephrology Unit, Department of Public Health, “Federico II” University, Naples, and the hospital outpatient clinic, “Federico II”, Naples. These patients were selected because they possessed truncating mutations in the *PKD1* and *PKD2* genes. Twelve out of the sixty-four patients were *PKD2* mutated [4]. The genetic characterization of ADPKD patients has been previously performed, and is shown in Supplementary Table S1 [4]. Blood samples were collected after a 12 h overnight fasting period and centrifuged to collect serum. Serum aliquots were immediately frozen in liquid nitrogen and stored at −80 °C. The following clinical and biochemical values were recorded (see Table 1): orexin-A, blood pressure, LDL, HDL, glucose, albumin, triglycerides, creatinuria, proteinuria, eGFR (CKD_epi formula was used to calculate eGFR), calcium, phosphorus, total cholesterol, and disease stage. The control group consisted of 24 healthy volunteers with an average age of 46 ± 9.67 years and a BMI of 24.82 ± 2.75. The local ethics committee authorized the research protocol, which was carried out in compliance with the guidelines of the Helsinki II Declaration. We obtained informed consent from each subject in accordance with Italian law.

### 4.2. ELISA Assay

A sandwich ELISA assay was used to measure orexin-A concentration in serum samples. The concentration of human OXA in the samples was determined by comparing the OD of the samples to the standard curve. This was measured spectrophotometrically at a wavelength of 450 nm ± 10 nm, according to the manufacturer’s instructions (Human OXA Elisa kit, ELK Biotechnology, Wuhan, China). Each sample was tested three times in duplicate. The Human OXA Elisa kit characteristics, as per the datasheet, demonstrate robust precision measures. Intra-assay precision exhibited a coefficient of variation (CV%) close to 8%, determined by testing three samples of known concentration twenty times on a single plate. Additionally, inter-assay precision boasted a CV% of less than 10%, determined by testing three samples across forty separate assays. The kit’s recovery was assessed by spiking serum with recombinant human OXA, yielding recovery rates ranging from 90% to 105%, with an average of 97%. For serum dilutions, the recovery rates were as follows: 1:2, 83–95%; 1:4, 91–101%; 1:8, 87–98%; and 1:16, 80–92%. Evaluation of linearity involved testing samples spiked with various concentrations of human OXA and their subsequent dilutions. Results were expressed as the percentage of calculated concentration compared to the expected concentration for the serum sample across different dilution ratios (1:2, 1:4, 1:8, and 1:16). In addition, this kit has high sensitivity and specificity for detection of human OXA, and no significant cross-reactivity or interference between human OXA and analogues was observed. (https://www.elkbiotech.com/pro/ELK1465, accessed on 1 March 2024).

### 4.3. SNPs: Sanger Sequencing

Using a resina illustraTM DNA extraction kit (GE Healthcare, Life Sciences, Chicago, IL, USA), genomic DNA was extracted from each blood sample. Applied Biosystems Inc., Foster City, CA, USA, manufactured the 2720 Thermal Cycler and the VeritiPro Thermal Cycler, which were used for PCR amplification. Subsequently, a 3730 DNA Analyzer (Applied Biosystems, Foster City, CA, USA) was used for direct sequencing. Supplementary Tables S2 and S3 display the polymorphisms for the HCRT, HCRTR1, and HCRTR2 genes. The Human Genome Variation Society (http://www.hgvs.org/mutnomen, accessed on 1 March 2024) standard gene variant nomenclature was followed, which was later updated by https://mutalyzer.nl. On request, amplification techniques and primers can be provided.

### 4.4. Statistical Analysis

The statistical analyses were conducted using GraphPad 6 Software, Inc. version 6.01 for Windows. The statistical significance threshold was set at *p* < 0.05, and the results were displayed as mean (M) ± standard deviation (SD). To verify that the variables had a normal distribution, the Shapiro–Wilk test was employed. An independent *t*-test was used to examine the variations in anthropometric measurements between healthy individuals and ADPKD patients. Additionally, an independent *t*-test was used to examine the variations in orexin-A levels between healthy individuals and ADPKD patients. Lastly, a linear regression analysis was run to look into the connections between orexin-A and eGFR and blood pressure, respectively.

## Figures and Tables

**Table 1:** Biochemical and clinical features of the study participants.

**Parameters**	**Healthy Subjects (n = 24)**	**PKD Patients (n = 64)**	***p*-Value**
Age	46.83 ± 9.46	43.58 ± 13.19	n.s
Sex	12 F/12 M	36 F/28 M	n.s
BMI	24.82 ± 2.75	24 ± 6.3	0.863
Orexin-A (pg/mL)	321.49 ± 78.01	477.07 ± 70.51	<0.0001
Systolic blood pressure (mm/Hg)	122.76 ± 7.73	134.45 ± 15.14	0.009
Diastolic blood pressure (mm/Hg)	82.92 ± 3.14	82.18 ± 7.4	0.732
Ca tot (mg/dL)	9.5 ± 0.41	9.65 ±0.56	0.283
Phosphorus (mg/dL)	3.62 ± 0.35	3.41 ± 4.48	0.107
Tot cholesterol (mg/dL)	205.86 ± 39.73	188.80 ± 35.14	0.063
Triglycerides (mg/dL)	122.17 ± 66.44	106.96 ± 54.61	0.286
LDL (mg/dL)	128.65 ± 29.88	116.67 ± 37.44	0.147
HDL (mg/dL)	54 ± 15.75	53.24 ± 19.01	0.859
Glucose (mg/dL)	85.17 ± 13.02	80.29 ± 14.01	0.220
Albumin (g/dL)	4.61 ± 0.45	4.54 ± 0.28	0.355
Creatinuria (mg/dL)	-	63.44 ± 30.3	-
Proteinuria (mg/dL)	-	39.78 ± 87.8	-
eGFR (mL/min/1.73 mq)	-	67.8 ± 34.53	-
**Chronic Kidney Disease (CKD)—Stage**		(%)	
G1	-	32.5	-
G2	-	21.6	-
G3a	-	17.6	-
G3b	-	17.6	-
G4	-	8.6	-
G5	-	2.1	-

**Table 2:** SNPs in the *HCRT* gene found in three ADPKD patients.

Patient Code	*HCRT* SNPs	Orexin-A (pg/mL)	eGFR (mL/min/1.73 mq)	Systolic Blood Pressure (mm/Hg)	Diastolic Blood Pressure (mm/Hg)
MPR 2023/22	rs9902709	456.29	58	130	80
ADPKDRR	rs9902709	440.59	15	150	90
MPR 2021/42	rs9902709	477.47	110	140	80

## Data Availability

Data is contained within the article and Supplementary Materials.

## Supplementary Materials

The following supporting information can be downloaded at: https://www.mdpi.com/article/10.3390/ijms25116243/s1.
