# Supplementary material for: Increased Expression of Orexin-A in Patients Affected by Polycystic Kidney Disease
Source: Int J Mol Sci. 2024 Jun 5;25(11):6243. doi: 10.3390/ijms25116243 (PMC11172798; doi:10.3390/ijms25116243)
Supplement: Supplementary file 1 [file ijms-25-06243-s001.zip › ijms-2979393-supplementary.pdf]

Supplementary Table S1. *PKD1* and *2* variants of patients.

PKD gene	cDNA change	Region	Protein variant	ACMG Classification	Coding impact
	c.10026delT	Exon 30	p.Leu3343SerfsTer54	P	frameshift
	c.10549G>T	Exon 35	p.Glu3517Ter	LP	nonsense
	C.10722G>A	Exon 36	p.Trp3574Ter	P	nonsense
	c.12058C>T	Exon 44	p.Arg4021Ter	P	nonsense
	c.3067C>T	Exon 13	p.Gln1023Ter	P	nonsense
	c.3514C>T	Exon 15	p.Gln1172Ter	P	nonsense
	c.4888C>T	Exon 15	p.Gln1630*	P	nonsense
	c.5905G>T	Exon 15	p.Glu1969Ter	P	nonsense
	c.8698C>T	Exon 23	p.Gln2900Ter	P	nonsense
	c.10420C>T	Exon 34	p.Gln3474Ter	P	nonsense
	c.10459C>T	Exon 34	p.Gln3487Ter	P	nonsense
	c.10722G>A	Exon 7	p.Trp3574Ter	P	nonsense
	c.10894_10895del	Exon 37	p.Ser3632ProfsTer88	P	frameshift
	c.1105_1106delAG	Exon 5	p.Ser369Ter	P	nonsense
	c.11438_11439delAT	Exon 41	p.Tyr3813Ter	LP	nonsense
	c.11571C>G	Exon 42	p.Tyr3857Ter	P	nonsense
	c.11646_11659del	Exon 42	p.Ser3883CysfsTer72	P	frameshift
	c.11705_11708delCCTC	Exon 42	p.Thr3902ArgfsTer41	LP	frameshift
	c.11881C>T	Exon 43	p.Gln3961Ter	P	nonsense
	c.11967_11974dup	Exon 43	p.Ser3992TrpfsTer49	P	frameshift
	c.12008dupA	Exon 44	p.Gln4005AlafsTer152	P	frameshift
	c.12058C>T	Exon 44	p.Arg4020Ter	P	nonsense
PKD1	c.12908A>T	Exon 46	p.4303LeuExontTer35	VUS	stopLoss
	c.2085delC	Exon 10	p.Ala696ArgfsTer89	P	frameshift
	c.2215dupC	Exon 11	p.Gln739ProfsTer59	LP	frameshift
	c.2711_2712delAG	Exon 11	p.Glu904GlyfsTer196	LP	frameshift
	c.3349C>T	Exon 15	p.Gln1117Ter	P	nonsense
	c.3398_3399delTG	Exon 15	p.Val1133GlufsTer2	LP	frameshift
	c.3520_3527delCAGCCGGC	Exon 15	p.Gln1174Cysfs34Ter	LP	frameshift
	c.3706C>T	Exon 15	p.Gln1236Ter	P	nonsense
	c.3745delG	Exon 15	p.Asp1249ThrfsTer24	P	frameshift
	c.3802C>T	Exon 15	p.Gln1268Ter	LP	nonsense
	c.427C>T	Exon 4	p.Gln143Ter	LP	nonsense
	c.4888C>T	Exon15	p.Gln1630Ter	P	nonsense
	c.4951C>T	Exon 15	p.Gln1651Ter	P	nonsense
	c.5154_5163dupGGGGTGGCTG	Exon 15	p.Met1722GlyfsTer52	P	frameshift
	c.5869_5870dupAG	Exon 15	p.Ser1957ArgfsTer16	LP	frameshift
	c.5884C>T	Exon 15	p.Gln1962Ter	P	nonsense
	c.6199C>T	Exon15	p.Gln2067Ter	P	nonsense
	c.7416_7417insC	Exon18	p.Gly2473ArgfsTer28	LP	frameshift
	c.7984C>T	Exon 21	p.Gln2662Ter	LP	nonsense
	c.8238delG	Exon 23	p.Met2747TrpfsTer9	LP	frameshift
	c.8371_8372dupCG	Exon 23	p.Ser2792GlyfsTer84	P	frameshift
	c.9425_9426insA	Exon 27	p.Tyr3143ValfsTer36	P	frameshift
	c.9771_9774delCTTT	Exon 29	p.Phe3257LeufsTer58	P	frameshift

	c.2614 C>T	Exon 14	p.Arg872Ter	LP	nonsense
	c.261G>A	Exon 1	p.Trp87Ter	P	nonsense
	c.916C>T	Exon 4	p.Arg306Ter	p	nonsense
	c.1395T>A	Exon 6	p.Tyr465Ter	LP	nonsense
PKD2	c.1837C>T	Exon 8	p.Gln613Ter	P	nonsense
	c.2358delG	Exon 12	p.Glu787ArgfsTer14	P	splicing
	c.2419C>T	Exon13	p.Arg807Ter	P	nonsense
	c.637C>T	Exon 2	p.Arg213Ter	p	nonsense
	c.958C>T	Exon 4	p.Arg320Ter	P	nonsense

Supplementary Table S2. *HCRT* analyzed SNPs.

**SNPs *HCRT* gene**	**cDNA**
**rs9902709**	c.21+16T>C
**rs1478595312**	c.158T>G
**rs761104247**	c.161A>G
**rs767907538**	c.163G>A
**rs1388119874**	c.167C>T
**rs1202994832**	c.169G>T
**rs769028179**	c.184G>A
**rs769028179**	c.184G>A
**rs763215233**	c.196C>T
**rs1415661572**	c.194C>G
**rs1438252070**	c.141C>G
**rs1010027499**	c.125A>G
**rs754232977**	c.114C>G
**rs754595506**	c.112G>A
**rs1373951353**	c.109C>T
**rs1418502204**	c.151G>C
**rs766682739**	c.153G>C

Supplementary Table S3. *HCRT1* and *2* analyzed SNPs.

**SNPs *HCRTR1* gene NM_001525.3**	**cDNA**
**rs2271933**	c.1222A>G
**SNPs *HCRTR2* gene NM_001526.5**	**cDNA**
**rs2653349**	c.922A>C/G
